# University of Louisville

**Published:** 1855-03

**Authors:** 


### UNIVERSITY OF LOUISVILLE.

The eighteenth session of the medical department of the University of Louisville was closed on the 2d of March by a public commencement, at which the degree of *Doctor of Medicine* was conferred upon seventy-two of the alumni. An appropriate address was delivered to the graduates by Professor J. L. Smith, who showed in his carefully prepared valedictory that to the skill and science of the chemist, he was capable of adding the grace of a polished diction and an impressive delivery. The size of the classes in this school for several years past has varied but little, and, notwithstanding the pecuniary pressure which materially reduced the number of medical students in the country, the class of last winter experienced no diminution.

